# Study on proximal humerus evaluation of effective treatment (SPHEER) – what is the effect of rehabilitation compliance on clinical outcomes of proximal humerus fractures

**DOI:** 10.1186/s12891-023-06894-w

**Published:** 2023-10-02

**Authors:** Ming Foo Kenneth Nah, Michelle Jessica Pereira, Mani Hemaavathi, Shiyun Winnie Wong, Chien Joo Lim, Bryan Yijia Tan

**Affiliations:** 1 Department of Orthopaedic Surgery, Tan Tock Seng Hospital, Singapore, Singapore; 2 Health Services and Outcomes Research, National Healthcare Group, Singapore, Singapore; 3 Nanyang Technological University, Singapore, Singapore; 4 Department of Occupational Therapy, Tan Tock Seng Hospital, Singapore, Singapore; 5 Medical Statistician (Research), Woodlands Health, Singapore, Singapore; 6 Department of Orthopedic Surgery, Woodlands Health, Singapore, Singapore

**Keywords:** Proximal humerus fracture, Prospective cohort study, Rehabilitation, Outcome measures, Compliance

## Abstract

**Introduction:**

Proximal humeral fractures (PHFs) are 3^rd^ commonest fragility fractures and cause significant functional impairment. This paper sought to determine impact of rehabilitation compliance on the clinical outcomes for non-surgically managed PHFs, while ascertaining reasons for non-compliance which can be addressed to improve compliance.

**Methods:**

Prospective cohort study of patients undergoing non-surgical treatment for PHFs from August 2017 to April 2020 in a tertiary trauma centre was performed. Data was collected via questionnaire: patient demographic data, PHF injury details, clinical outcome measures, therapist-reported (Sport Injury Rehabilitation Adherence Scale [SIRAS]) and patient-reported (subjective compliance, frequency of exercise) rehabilitation compliance measures. Data was analysed using multiple linear regression model to account for confounding variables.

**Results:**

107 participants attended physical therapy follow-up for mean 137.8 days. 6-week SIRAS strongly predicted 3-month Constant score (*p* = 0.023; 95%CI = 0.265,3.423), OSS (*p* = 0.038; 95%CI = 0.049,1.634), flexion ROM (*p* < 0.001; 95%CI = 2.872,8.982), extension ROM (*p* = 0.035; 95%CI = 0.097,2.614), abduction ROM (*p* = 0.002;95%CI = 1.995,8.466) and achievement of functional active ROM at 3-months (*p* = 0.049; 95%CI = 1.001,1.638). Pain was the top reason impairing rehabilitation compliance from therapist (43.9% at 6-weeks and 20.6% at 3-months) and patient-perspective (33.6% at 6-weeks, 24.3% at 3-months). Author-developed patient-reported compliance measures had good correlation with validated SIRAS score (subjective compliance: *p* < 0.001 frequency of exercise: *p* = 0.001).

**Conclusion:**

Rehabilitation compliance predicts short-term clinical outcomes up to 3-months and potentially 1-year outcomes. Pain control should be optimised to maximise rehabilitation compliance and improve PHF outcomes. There is lack of consensus definition for rehabilitation compliance measures; patient-reported measures used have good correlation to existing validated measures and could serve as a steppingstone for further research.

**Level of evidence:**

II, cohort study.

**Supplementary Information:**

The online version contains supplementary material available at 10.1186/s12891-023-06894-w.

## Key Points

Findings: Good rehabilitation compliance predicts improved clinical outcomes in non-surgically managed PHFs, with stronger predictive value on short-term outcomes at 3 months compared to long-term outcomes at 1 year. Patient-reported rehabilitation compliance measures show good promise for the evaluation of rehabilitation compliance.

Implications: Focus should be made to optimise analgesia and minimizing pain for conservatively managed PHFs so that rehabilitation compliance can be improved and contribute to better clinical outcomes. There should be further research to validate the author-proposed patient-reported rehabilitation compliance measures within this study. This can be expanded to other musculoskeletal injuries on top of PHFs as well.

Caution: The accuracy of measures of rehabilitation compliance in this study may be slightly limited in view of 1) no longer followed up after discharged from occupational therapy and 2) the author-developed patient-reported measures, while promising, are not fully validated for PHF outcomes as well.

**Supplementary Information:**

The online version contains supplementary material available at 10.1186/s12891-023-06894-w.

## Introduction

Proximal humerus fractures (PHFs) account for a significant fraction of fragility fractures, comprising 5–6% of all adult fractures [1], with higher preponderance for osteoporotic elderly females [2]. PHFs are associated with substantial morbidity and impaired activities of daily living (ADLs) for upwards of 2–3 months [3–5]. As such, minimising functional impairment is an important goal of PHF treatment.

Treatment of PHFs can be non-surgical or surgical (e.g. fixation, arthroplasty). The ideal treatment modality for PHFs is controversial as treatment choice is variable and based on multiple factors (e.g. fracture morphology, pre-morbid function, comorbidities precluding surgery) [6]. Ultimately, whichever management modality is chosen, treatment aims to facilitate return of upper limb function. This traditionally involves a comprehensive rehabilitation regime [7], beginning with immobilisation followed by exercises to maximise passive range of motion (PROM), active range of motion (AROM) and eventually progressive resistive/strengthening exercises [8–10]. Progression of therapy is prescribed by therapists in collaboration with surgeons, accounting for fracture healing/stability.

Rehabilitation compliance is associated with enhanced patient outcomes for other conditions such as stroke [11] and anterior cruciate ligament repairs [12]. Similar emphasis is placed on compliance to PHF rehabilitation. Yet, scoping reviews have shown that literature supporting relationships between rehabilitation compliance and better clinical outcomes in PHFs is lacking [13, 14]. Furthermore, certain studies even propose that rehabilitation compliance has no positive effect on functional outcomes in PHFs [15].

The primary aim of this study is to identify relationships between rehabilitation compliance and short-term (3-month)/long-term (1 year) clinical outcomes. The authors hypothesize that improved rehabilitation compliance would be associated with better short- and long-term clinical outcomes. A secondary aim is to establish reasons for rehabilitation non-compliance which can be addressed to improve compliance.

## Methods

### Study design

This is a prospective cohort study of patients in a Singaporean 1700-bedder tertiary trauma centre which sees a large volume of PHFs undergoing non-surgical management. Ethic clearance by the institution’s research governing board was obtained prior to any research-related activities. Study design was guided by the STrengthening the Reporting of OBservational Studies in Epidemiology (STROBE)”, which was created to aid authors in ensuring high-quality presentation of the conducted observational studies (Appendix 1).

### Patient population

The study sample was derived by applying inclusion/exclusion criteria (Table 1) to the cohort of PHF patients undergoing non-surgical treatment from 21 August 2017 to 1 April 2020. These criteria were picked to optimise homogeneity of the study sample (e.g. excluding polytrauma and open fractures) and ensure ability to participate in rehabilitation as per the institution’s PHF rehabilitation protocol (e.g. excluding those with late presentation > 3 weeks, without mental capacity). PHFs were defined as fractures proximal to humeral surgical neck, diagnosed via orthogonal shoulder radiographs (anteroposterior/Y-scapula views). Informed consent to document patient demographics and clinical data for research purposes was received and the rights of the subjects were protected via anonymity of data. All patients underwent rehabilitation under the institution’s standard rehabilitation protocol (Appendix 2) which progressed through phases of passive range of motion exercises, active range of motion exercises and progressive resistance training. Each patient’s programme was tailored to their progress, tolerance and functional demands.

**Table 1:** Inclusion and exclusion criteria

**Inclusion Criteria**
1. Radiographically proven closed proximal humerus fracture treated non-surgically^a^
2. > 21 years of age^b^
3. Acute fracture presenting within 3 weeks of injury
**Exclusion Criteria**
1. Surgical indications
a. Open fracture
b. Severe soft tissue compromise
c. Neurovascular injury
2. Confounding factors which may affect functional outcomes after rehabilitation
a. Multiple injuries
b. Pathological fractures
c. Patients without mental capacity
3. Anaesthetic issues that may affect decision for surgery
a. Pregnancy
b. Co-morbidities precluding anaesthesia

^a^Decision for non-surgical treatment was a joint decision made between the patients and the managing surgeon taking into account both injury, radiographic and patient factors

^b^21 years was used as a cut-off for participant consent

### Data collection

Data collection was performed using standardised questionnaire (Appendix 3) filled in by patients/occupational therapists at the same time junctures (initial therapy visit and 6-week /3-month/1-year post-injury reviews). Functional outcomes were recorded at 3-month and 1-year. Rehabilitation compliance measures were recorded at 6-week and 3-month. Each patient was chronologically assigned a patient number for anonymity.

### Patient demographics and injury details

Patient demographics was collected within the standardised questionnaires. Injury parameters such as involvement of dominant arm and Neer’s classificationwere also collected as these were potential confounding factors which have been shown to affect clinical outcomes of non-surgically managed PHFs [16, 17], and accounted for in subsequent statistical analysis.

### Clinical outcome measures

Given that there is no consensus / gold standard for outcome measures of PHF studies [18], the authors collected seven clinical outcome measures commonly used in PHF research to reflect upper limb function.

Firstly, affected shoulder AROM (flexion, extension, abduction, internal rotation, external rotation) was measured with BASELINE™ 12-1012HR goniometers by occupational therapists during rehabilitation sessions and served as objective measure of shoulder function. On top of individual ROM parameters, the achievement of functional shoulder AROM (i.e. minimum shoulder ROM required for activities of daily living (ADLs) – established as 115° flexion/40° extension/120° abduction/50° internal rotation/45° external rotation [19]) also reflected functional recovery/independence. Finger grip strength measured via JAMAR® Hydraulic Hand Dynamometers demonstrated upper limb muscle strength and has good correlation with validated upper limb functional scores such as DASH score [2, 20]. Pain scoring in the form of the well-validated NRS was also included.

Additionally, three composite scores validated to measure functional outcomes in non-surgically managed PHFs were recorded [21]. The QuickDASH score is an 11-item patient-reported score which reflects subjective clinical outcomes (disability and symptoms) [2]. It is an abbreviated and reliable adaption of the more comprehensive 30-item DASH score, helping minimise responder burden and maximise ease of scoring [2]. The Constant score is a 100 point-scale inclusive of 4 therapist-assessed/patient-reported domains – pain, mobility, strength and ability to cope with activities of daily living (ADLs) [21], while the Oxford Shoulder score (OSS) is a 12-item patient-reported score originally designed for assessing outcomes of shoulder surgery but shows good reliability and sensitivity to change over time for conservatively managed PHFs [21].

### Measures of rehabilitation compliance

There is no “perfect” measure of rehabilitation compliance for several reasons. Firstly, there are multiple aspects of compliance, each being more appropriate in different contexts [22]. For instance, attendance (%therapy sessions attended over total sessions offered) should be the key measure if the question is financial viability of a therapy programme [23]. If the research question focuses on functional outcomes, other compliance factors may bear more significance, e.g. duration of sessions, intensity of exercises (physiological/aerobic demand). Lastly, rehabilitation compliance is inherently subjective, what is deemed high intensity for one may not be high intensity for another.

Existing self-reported exercise adherence questionnaires tend to be lengthy and unvalidated [23]. To holistically evaluate rehabilitation compliance, the authors developed a short patient-/therapist-reported questionnaire to reduce responder burden, while still capturing domains commonly cited in rehabilitation compliance studies such as frequency and intensity [7]. This questionnaire was pilot tested prior to use to ensure accurate translation, comprehensibility and ease of completion.

Two patient-reported compliance measures were used – patient-reported subjective compliance in performing prescribed exercises (asked to “Rate [their] compliance in performing the exercises prescribed” with options ranging from “Not at all” to “All the time”) and frequency of average regime at home for exercises prescribed (asked “On average, what is [their] regime for the exercises prescribed?” with options ranging from “None at all” to “ > 3 sessions per day”). These responses were converted to numerical values on a scale of 0–5 (5 being the highest compliance) to allow for statistical analysis. These measures are author-developed and unvalidated. A 5-point Likert Scale was used as it is shown to improve response rate/quality and reduce responders’ frustration levels [24]. It also allowed consistency with and comparisons to be made with the validated Sport Injury Rehabilitation Adherence Scale (SIRAS), which was also included in the questionnaire.

Therapist-reported rehabilitation compliance was based on the SIRAS, a well-validated scoring instrument for rehabilitation of musculoskeletal injuries with rater-agreement index values of up to 0.954 [25]. The SIRAS comprises3 questions concerning rehabilitation intensity, patients’ ability to follow instructions and patients’ receptivity to change in program. Each component is worth a best possible 5 points, adding up to a total of 15 [26].

Lastly, the questionnaire also sought the opinion of patients/therapists regarding factors that may hinder rehabilitation compliance to prescribed rehabilitation exercises.

### Statistical analysis

Data was cleaned/analysed using STATA version 14.0. Demographics/clinical outcomes were presented using descriptive statistics. Distribution of numerical data was assessed using histogram and presented using mean/standard deviation as the distribution was found to be approximately normal, while categorical variables were presented using frequency/percentage.

Multiple linear regression was used to identify relationships between rehabilitation compliance measures and clinical outcomes. This allowed the use of multiple explanatory variables in a model to predict outcome and helped control for potential confounders. The parameters shown to affect functional outcomes of non-surgically managed PHFs [16, 27] – age, gender, Neer’s classification, involvement of dominant hand, functional expectations (e.g. employment status) were considered. Assumptions of the multivariable models were checked, heteroskedasticity tested using Breusch-Pagan/Cook-Weisberg test and scatter plot of predicted values versus residuals. Spearman correlation was also used to explore strength of relationships between therapist-reported and patient-reported compliance measures given the ordinal nature of the variables. Statistical significance was denoted as *p* < 0.05.

## Results

One hundred seven patients were selected, and all completed study follow-up to 1-year post-fracture. Demographic data is summarised in Table 2, with mean age of 69.46 years, 79.4% female and 96.3% right-handed. There was no pattern to side of injury/involvement of dominant hand, and majority of PHFs were Neer’s 2-part (32.7%)/3-part (30.8%).

**Table 2:** Demographic characteristics of the participants (*n* = 107) as obtained from questionnaire

		**Mean (SD**^**a**^**)**
**Age / years**		69.46
**Duration before discharge from regular therapy sessions / days**		137.86
**Gender, n (%)**	Male	22 (20.6)
	Female	85 (79.4)
**Hand Dominance, n (%)**	Left	4 (3.7)
	Right	103 (96.3)
**Side of Injury, n (%)**	Left	50 (46.7)
	Right	57 (53.3)
**Dominant hand affected, n (%)**	No	48 (44.9)
	Yes	59 (55.1)
**Employment status, n (%)**	Employed	36 (33.6)
	Homemaker	26 (24.3)
	Unemployed	2 (1.9)
	Retired	43 (40.2)
**Neer’s classification, n (%)**	1-part	21 (19.6)
	2-part	35 (32.7)
	3-part	33 (30.8)
	4-part	17 (15.9)

^a^*SD* Standard Deviation

**Fig. 1:**
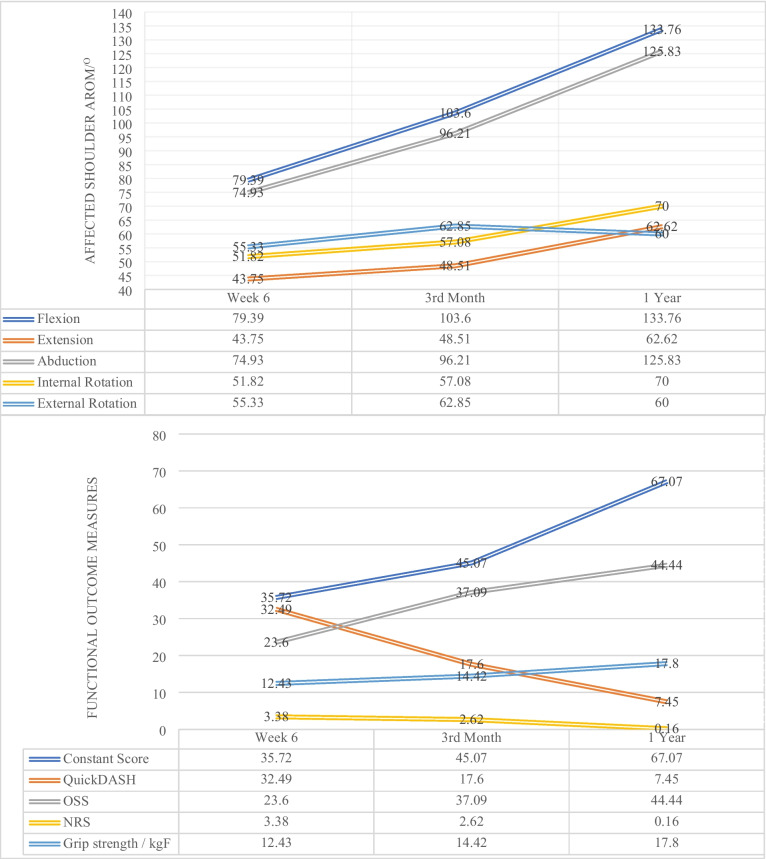
Graphical representation of clinical outcome measures over time

Figure 1 illustrates the improvement of all clinical outcome measures over time, except for shoulder external rotation, which improved from 55.33° at 6 weeks to 62.85° at 3 months but fell to 60° at 1 year. 34/107 patients (31.78%) achieved functional shoulder AROM at 3-month, while 84/107 patients (78.50%) did so at 1-year.

**Fig. 2:**
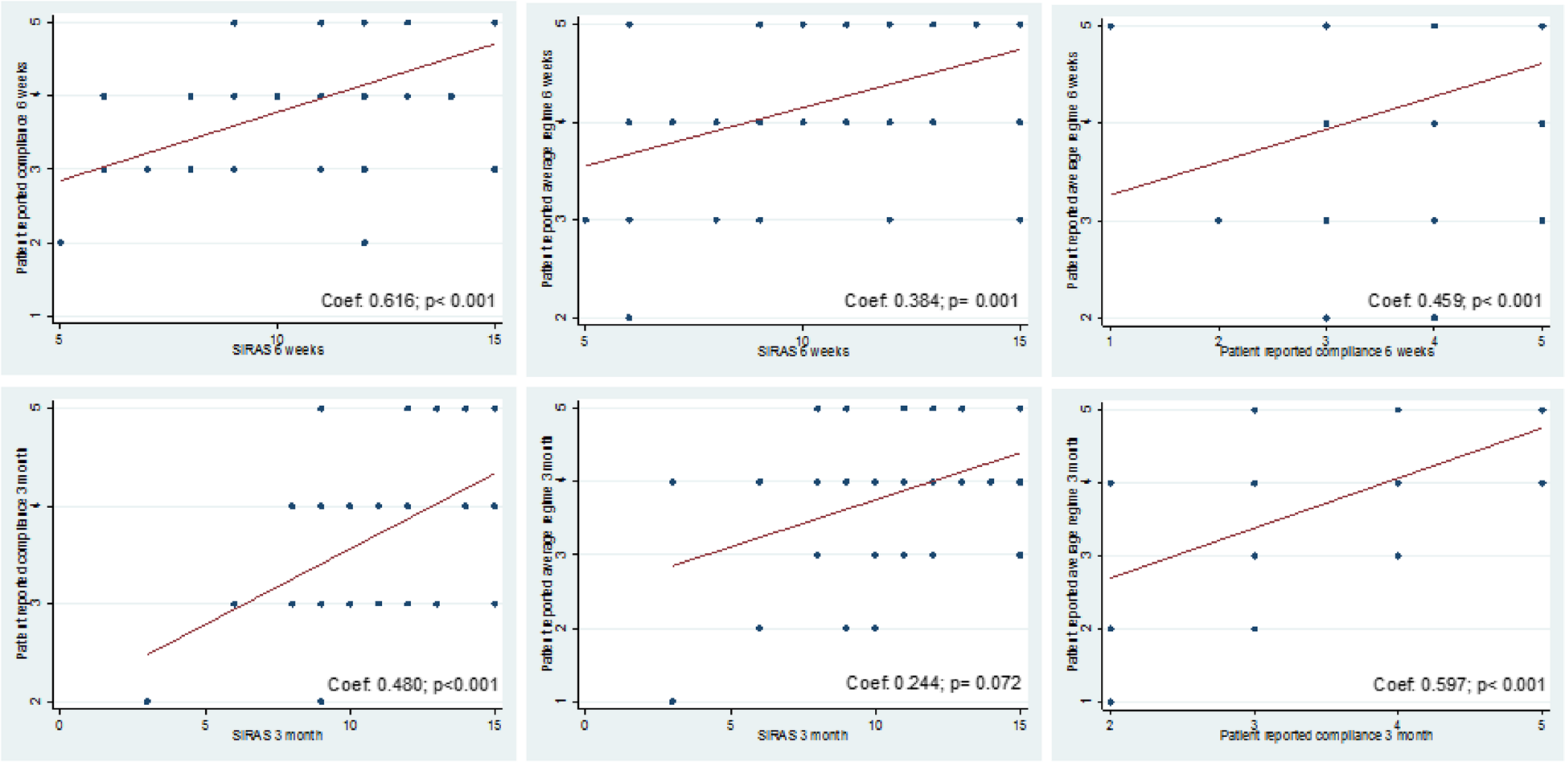
Correlation between rehabilitation compliance measures

Table 3 shows the subjective patient- and therapist-reported compliance measures. Patients reported mean subjective compliance scores of 3.93/5 at 6-weeks post-injury, which reduced to 3.69/5 at the 3-months post-injury. A similar pattern is seen for the patient-reported average regime in prescribed exercises with a mean score of 4.25/5 at 6 weeks and 3.86/5 at 3 months. Likewise, patients had an average SIRAS score (therapist-reported) of 1061/15 at 6 weeks and 1039/15 at 3 months. The two self-developed patient-reported rehabilitation compliance measures had significant correlation with the well-validated SIRAS. As shown in Fig. 2, 6-week patient subjective compliance (*p* < 0.001, *r* = 0.616) and 6-week patient-reported average regime (*p* = 0.001,r = 0.384) were moderately correlated with 6-week SIRAS while the 3-month patient subjective compliance (*p* < 0001,*r* = 0.480) was moderately correlated to the 3-month SIRAS. However, 3-month patient-reported average regime (*p* = 0.072, *r* = 0.244) was not significantly correlated to the 3-month SIRAS.

**Table 3:** Subjective (Therapist-reported and Patient-reported) and objective rehabilitation compliance measures as obtained from questionnaire

Rehabilitation compliance measures	Week 6	3^rd^ Month
Therapist Reported		
Intensity, mean (SD^b^)	3.42 (0.97)	3.34 (0.98)
Instructions, mean (SD^b^)	3.56 (0.95)	3.47 (0.98)
Receptive, mean (SD^b^)	3.65 (0.91)	3.59 (1.01)
SIRAS^a^, mean (SD^b^)	10.61 (2.66)	10.40 (2.75)
Patient Reported		
Subjective compliance in performing exercise prescribed, mean (SD^b^)	3.93 (0.84)	3.69 (0.82)
Average regime for the exercise prescribed, mean (SD*)	4.25 (0.69)	3.86 (0.93)

^a^*SIRAS* Sport Injury Rehabilitation Adherence Scale

^b^*SD* Standard Deviation

Tables 4 and 5 depict results from multiple linear regression analysis performed between rehabilitation compliance measures and clinical outcome measures – with significant results bolded. Rehabilitation compliance in the first 6-week to 3-month period predict improved clinical outcomes, predominantly for short-term clinical outcomes (3-month), although still witnessed in the long-term (1-year). Achieving functional shoulder AROM at 3-month is predicted by higher 6-week SIRAS (*p* = 0.049,adj. OR = 1.280) and 3-month patient subjective compliance (*p* = 0.009,adj. OR = 3.503) but there was no significant predictive relationship for achieving functional shoulder ROM at 1-year.

**Table 4:** Regression analysis between rehabilitation compliance measures and composite clinical scores

3 Months									
	**QuickDASH**^**c**^			**Constant**			**OSS**^**d**^		
	**Adj. coef**^**a**^	**95% CI**^**b**^	***p* value**	**Adj. coef**^**a**^	**95% CI**^**b**^	***p* value**	**Adj. coef**^**a**^	**95% CI**^**b**^	***p* value**
**6-week compliance measures**									
SIRAS^e^	-0.784	-2.734, 1.166	0.421	1.844	0.265, 3.423	**0.023**	0.842	0.049, 1.634	**0.038**
Patient Subjective compliance	-0.702	-4.840, 3.436	0.736	1.770	-1.738, 5.278	0.318	1.920	0.099, 3.742	**0.039**
Patient-reported Average Rehab Regime	-1.999	-7.287, 3.290	0.453	1.500	-3.035, 6.034	0.511	0.197	-2.195, 2.589	0.870
**3-month compliance measures**									
SIRAS^e^	-0.491	-2.648, 1.665	0.647	1.085	-0.269, 2.440	0.113	0.852	-0.046, 1.751	0.063
Patient Subjective compliance	0.527	-4.658, 5.711	0.840	2.661	-1.024, 6.346	0.154	1.159	-1.326, 3.645	0.355
Patient-reported Average Rehab Regime	1.602	-2.795, 5.998	0.469	0.085	-3.000, 3.170	0.956	0.578	-1.496, 2.652	0.580
**1 Year**									
	**QuickDASH**^**c**^			**Constant**			**OSS**^**d**^		
	**Adj. coef**^**a**^	**95% CI**^**b**^	***p* value**	**Adj. coef**^**a**^	**95% CI**^**b**^	***p* value**	**Adj. coef**^**a**^	**95% CI**^**b**^	***p* value**
**6-week compliance measures**									
SIRAS^e^	-0.059	-0.210, 0.092	0.427	1.192	-0.352, 2.736	0.123	-0.001	-0.011, 0.010	0.872
Patient Subjective compliance	-0.191	-0.530, 0.149	0.259	1.704	-1.374, 4.781	0.267	0.002	-0.019, 0.023	0.856
Patient-reported Average Rehab Regime	-0.313	-0.823, 0.197	0.218	-0.033	-4.256, 4.191	0.988	-0.023	-0.050, 0.005	0.102
**3-month compliance measures**									
SIRAS^e^	-0.002	-0.167, 0.164	0.984	0.600	-0.677, 1.876	0.337	-0.001	-0.011, 0.009	0.861
Patient Subjective compliance	-0.016	-0.521, 0.489	0.948	-0.090	-4.498, 4.319	0.967	0.006	-0.021, 0.034	0.651
Patient-reported Average Rehab Regime	0.013	-0.395, 0.422	0.946	-2.365	-5.846, 1.117	0.174	0.002	-0.022, 0.025	0.888

Significant results are bolded

^a^*Adj. coef* Adjustment coefficient (unstandardised)

^b^*CI* Confidence interval

^c^*QuickDASH* Quick Disability of Arm, Shoulder, Hand

^d^*OSS* Oxford Shoulder Score

^e^*SIRAS* Sport Injury Rehabilitation Adherence Scale

**Table 5:** Results from regression analysis comparing rehabilitation compliance measures and affected shoulder ROM measurements

3 Months									
	**Flexion**			**Extension**			**Abduction**		
	**Adj. coef**^**a**^	**95% CI**^**b**^	***p* value**	**Adj. coef**^**a**^	**95% CI**^**b**^	***p* value**	**Adj. coef**^**a**^	**95% CI**^**b**^	***p* value**
**6-week compliance measures**									
SIRAS^c^	5.912	2.872, 8.982	** < 0.001**	1.355	0.097, 2.614	**0.035**	5.231	1.995, 8.466	**0.002**
Patient Subjective compliance	9.189	2.034, 16.344	**0.013**	1.147	-1.960, 4.254	0.465	6.625	-1.433, 14.683	0.106
Patient-reported Average Rehab Regime	5.089	-4.107, 14.284	0.274	0.683	-3.191, 4.557	0.727	2.430	-7.759, 12.609	0.636
**3-month compliance measures**									
SIRAS^c^	4.807	1.884, 7.731	**0.002**	1.434	0.192, 2.675	**0.025**	4.457	1.320, 7.594	**0.006**
Patient Subjective compliance	14.704	6.450, 22.958	**0.001**	3.158	-0.590, 6.906	0.097	12.411	3.595, 21.228	**0.006**
Patient-reported Average Rehab Regime	6.086	-1.214, 13.385	0.101	0.313	-2.846, 3.471	0.844	2.075	-5.629, 9.778	0.593
	**Internal Rotation**			**External Rotation**			**Achieving functional AROM**^**d**^		
	**Adj. coef**^**a**^	**95% CI**^**b**^	***p* value**	**Adj. coef**^**a**^	**95% CI**^**b**^	***p* value**	**Adj. coef**^**a**^	**95% CI**^**b**^	***p* value**
**6-week compliance measures**									
SIRAS^c^	-0.795	-3.124, 1.534	0.495	1.081	-0.808, 2.970	0.255	1.280	1.001, 1.638	**0.049**
Patient Subjective compliance	-1.825	-6.904, 3.254	0.476	1.228	-3.370, 5.826	0.596	1.2670	0.742, 2.162	0.386
Patient-reported Average Rehab Regime	-1.581	-7.870, 4.707	0.618	-2.383	-8.051, 3.284	0.405	1.018	0.515, 2.010	0.959
**3-month compliance measures**									
SIRAS^c^	-0.265	-2.310, 1.811	0.798	2.597	0.791, 4.403	***0.006***	1.477	0.937, 2.327	0.093
Patient Subjective compliance	0.260	-5.371, 5.890	0.927	4.511	-0.534, 9.556	0.079	3.503	1.363, 9.004	**0.009**
Patient-reported Average Rehab Regime	0.924	-3.945, 5.793	0.706	0.075	-4.388, 4.539	0.973	1.439	0.717, 2.885	0.306
**1 Year**									
	**Flexion**			**Extension**			**Abduction**		
	**Adj. coef**^**a**^	**95% CI**^**b**^	***p* value**	**Adj. coef**^**a**^	**95% CI**^**b**^	***p* value**	**Adj. coef**^**a**^	**95% CI**^**b**^	***p* value**
**6-week compliance measures**									
SIRAS^c^	6.823	3.399, 10.247	**0.001**	2.402	0.274,4.529	**0.029**	5.546	0.909, 10.183	**0.021**
Patient Subjective compliance	4.567	-5.473, 14.606	0.361	2.454	-1.724, 6.631	0.240	5.422	-5.201, 16.046	0.307
Patient-reported Average Rehab Regime	8.964	-4.362, 22.291	0.180	2.479	-3.084, 8.042	0.371	5.545	-8.867, 19.956	0.439
**3-month compliance measures**									
SIRAS^c^	3.063	-0.861, 6.988	0.118	1.501	-0.471, 3.473	0.128	2.901	-1.778, 7.580	0.210
Patient Subjective compliance	2.038	-12.971, 17.047	0.783	-0.134	-7.064, 6.797	0.969	-2.543	-17.995, 12.91	0.738
Patient-reported Average Rehab Regime	8.964	-4.362, 22.291	0.180	-0.651	-6.382, 5.079	0.817	-6.427	-18.979, 6.124	0.303
	**Internal Rotation**			**External Rotation**			**Achieving functional AROM**^**d**^		
	**Adj. coef**^**a**^	**95% CI**^**b**^	***p* value**	**Adj. coef**^**a**^	**95% CI**^**b**^	***p* value**	**Adj. coef**^**a**^	**95% CI**^**b**^	***p* value**
**6-week compliance measures**									
SIRAS^c^	0.282	-1.813, 2.377	0.782	0.452	-2.573, 3.477	0.758	1.090	0.855, 1.390	0.487
Patient Subjective compliance	-1.607	-5.646, 2.431	0.424	-1.503	-6.720, 3.714	0.562	1.133	0.633, 2.025	0.675
Patient-reported Average Rehab Regime	-0.069	-5.563, 5.425	0.980	-1.870	-8.907, 5.164	0.592	0.959	0.446, 2.064	0.915
**3-month compliance measures**									
SIRAS^c^	0.778	-1.108, 2.664	0.399	-0.366	-3.370, 2.637	0.801	0.870	0.676, 1.120	0.280
Patient Subjective compliance	-2.543	-8.474, 3.388	0.387	1.317	-5.892, 8.525	0.711	0.831	0.406, 1.701	0.613
Patient-reported Average Rehab Regime	-3.228	-8.036, 1.581	0.180	0.385	-5.589, 6.360	0.896	0.529	0.254, 1.100	0.088

Significant results are bolded

^a^*Adj. coef* Adjustment coefficient (unstandardised)

^b^*CI* Confidence interval

^c^*SIRAS* Sport Injury Rehabilitation Adherence Scale

^d^*AROM* Active range of motion

In addition, 6-week SIRAS showed significant predictive relationships with multiple 3-month but only 3 1-year clinical outcome measures, predicting 3-month Constant Score (*p*-value = 0.023,adj. coef = 1.844), 3-month OSS (*p*-value = 0.038,adj. coef = 0.842), 3-month flexion (*p*-value < 0.001,adj. coef = 5.912), 3-month extension (*p*-value = 0.035,adj. coef = 1.355), 3-month abduction (*p*-value = 0.002,adj. coef = 5.231), achieving function AROM at 3-month (*p*-value = 0.049,adj. coef = 1.280) on top of 1-year flexion (*p*-value = 0.001,adj. coef = 6.823), 1-year extension (*p*-value = 0.029,adj. coef = 2.492) and 1-year abduction (*p*-value = 0.021,adj. coef = 5.546). A direct comparison between 3-month and 1-year clinical outcomes also shows that 6-week SIRAS has stronger predictive value for short-term outcomes. 6-week SIRAS has a better predictive relationship with 3-month Constant score than 1-year Constant score (*p*-value = 0.023 vs 0.123). This is also seen for OSS (0.038 vs 0.872), flexion (< 0.001 vs 0.002), abduction (0.002 vs 0.006) and achieving function AROM (0.049 vs 0.093).

None of the rehabilitation compliance measures predicted short or long-term QuickDASH and NRS. These rehabilitation compliance measures also poorly predict finger grip strength – with only grip strength at 1-year being predicted by 6-week SIRAS (*p* = 0.012,adj coef. = 1.621).

Lastly, Table 6 summarises patient/therapist-reported reasons for non-compliance. The authors used frequency and number of patients affected to rank the factors. Pain was consistently the top reason for non-compliance from both therapist and patient perspectives – at both 6-week and 3-month marks. Other commonly cited reasons from therapist-perspective included impaired cognitive learning ability and time constraints while patients reported time constraints and forgetfulness as other contributing factors towards non-compliance.

**Table 6:** Secondary outcomes – results for therapist-reported and patient-reported reasons for non-compliance as collated from the questionnaire

	**Week 6**	**3**^**rd**^** Month**
**Therapist Reported**		
Factors affecting patient’s compliance in home program, frequency (%)		
Pain guarding	**47 (43.9)**	**22 (20.6)**
Fear of re-injury	15 (14.0)	9 (8.4)
Lack of interest/ motivation	8 (7.5)	10 (9.3)
Time constraint/ Return to work	15 (14.0)	18 (16.8)
Learning ability	22 (20.6)	19 (17.8)
Cognitive	12 (54.55)	9 (47.37)
Language	0 (0.0)	0 (0.0)
Physical ability	11 (50.0)	12 (63.16)
Defaulted therapy	2 (1.9)	2 (1.9)
Others	19 (17.8)	30 (28.0)
**Patient Reported**		
Reasons for not complying with exercises prescribed, frequency (%)		
Unsure of the exercises to do	6 (5.6)	5 (4.7)
Time constraint	9 (8.4)	20 (18.7)
Pain	**36 (33.6)**	**26 (24.3)**
Need assistance from others	9 (8.4)	8 (7.5)
Financial	0 (0.0)	0 (0.0)
Forgetful	17 (15.9)	15 (14.0)
Do not believe that therapy will work	1 (0.9)	0 (0.0)
Others	24 (22.4)	25 (23.4)

Significant results are bolded

## Discussion

This study aimed to identify relationships between rehabilitation compliance and short/long-term clinical outcomes for non-surgically managed PHFs, and identify factors contributing to non-compliance.

### Improved short-term and long-term clinical outcomes

Rehabilitation compliance had stronger correlation with short-term compared to long-term clinical outcomes – with significant predictive relationships seen between 6-week rehabilitation compliance measures and 3-month Constant score, OSS and shoulder AROM. Results showed that therapist-reported compliance (SIRAS) at 6-weeks significantly predicted 3-month Constant score (*p* = 0.023) and OSS (*p* = 0.038), while 6-week patient subjective compliance significantly predicted 6-week OSS (*p*= 0.039). There was no such relationship between rehabilitation compliance measures and 1-year outcome measures. Furthermore, head-to-head comparison between multiple 3-month outcome measures (Constant score/OSS/flexion/ abduction/achieving functional AROM) showed that rehabilitation compliance tends to predict 3-month outcomes better than the respective 1-year counterparts. A postulation for this is that the patient cohort – typically discharged from therapy when deemed to have achieved functional goals based on their functional expectations and requirements – tends to see a drop in exercise compliance and effort after discharge. This psychology towards rehabilitation is well documented in existing literature, as patients tend to work harder/perform longer when there is a concrete goal to provide purpose and serve as a distraction from the effort required. This may be true for short-term rehabilitation where patients seek to regain function and improve between clinic-based therapy sessions. However, the purpose of continued long-term post-discharge therapy is usually the maintenance of function – which may be comparatively poor motivation as there is no concrete end-point and patients may find it difficult to perceive any benefits [28]. Given that the mean duration of therapy follow-up for our patient cohort (standardized as number of days between date of injury and discharge from therapist follow-up) was 137.86 days, or roughly 4.5 months, this could explain the lack of positive correlation for rehabilitation compliance and longer-term clinical outcomes.

In addition, a 2011 systemic review by Bruder et al. of trials exploring impact of exercise/rehabilitation on functional outcomes in upper limb fractures has shown that evidence on the adherence/compliance in PHF rehabilitation is lacking [29]. Only 1/13 trials in this review reported some form of “adherence” to an exercise program for PHF patients. This was an RCT of 74 patients by Lefevre-Colau et al. in 2007 that sought to compare clinical outcomes in PHFs treated by early rehabilitation (after 72 h of immobilisation) and delayed rehabilitation (after 3 weeks of immobilisation) – concluding that early rehabilitation improved functional outcomes and symptoms (e.g. pain) [30]. Adherence to rehabilitation regimes was only recorded as a secondary parameter to account for confounding factors of clinical outcomes, and only a single adherence/compliance measure was recorded (patients’ attendance of the exercise sessions). Otherwise, other PHF-related studies focused more on comparing different exercise interventions (e.g. home exercise programme alone versus a combination of home/supervised exercise sessions) rather than compliance to a rehabilitation regime. This study attempted to plug this gap in existing literature, incorporating a holistic set of rehabilitation compliance measures encompassing perspectives of main stakeholders (patient/therapist), to show how rehabilitation compliance can improve short-term and potentially long-term clinical outcomes.

The systemic review by Bruder et al. also highlighted multiple trials with a similar conclusion – that increased exercise/rehabilitation contributes to improved short-term reduction in impairment (with ROM/strength being commonly used parameters) [29]. However, few papers considered how improvements of these parameters translates to improvements in ability to complete daily functions. In this paper, this was addressed by including clinical outcome measures which reflect a patient’s ability to cope with ADLs – e.g. achieving functional AROM and 3 composite functional scores (Constant Score/OSS/QuickDASH). Given the relationship between short-term clinical outcomes and rehabilitation compliance, rehabilitation compliance (and its obstacles) should be viewed with utmost importance, especially for patients who are likely to benefit greatly from earlier return to functional independence, e.g. the employed or patients without caregivers. Such patients may even benefit from accelerated rehabilitation programs with higher intensity or frequency of sessions in the short-term period post-injury and from any interventions to address reasons for non-compliance, to facilitate regaining functional independence earlier.

### Tackling the obstacles to rehabilitation compliance

Given the importance of rehabilitation compliance for improved short-term outcomes and functional independence, efforts should be made to facilitate PHF rehabilitation compliance via establishing a strong social support network, reducing rehabilitation-related anxiety/stress via patient education or introducing motivational strategies [31].

In our sample, pain was the most frequently cited reason for non-compliance at 6-week and 3-month from both therapist and patient perspective within our sample. Currently, analgesia for PHF patients is largely limited to oral options as guided by the WHO pain ladder. However, there has been promising evidence for pain adjuncts in PHF rehabilitation. A large-scale systemic review by Iliaens et al. has shown that interscalene nerve blocks have had promising results for PHF patients, with decreased opioid requirements and improved functional scores (quickDASH, shoulder ROM) after rehabilitation [27]. Given that moderate-to-severe acute pain after major shoulder surgery tends to last 48 h post-operatively, different institutions have also adopted different modalities of regional anaesthesia for shoulder surgery, such as single-shot interscalene block (SISB) which can potentially provide effective analgesia for 8–72 h [6, 27]. This similar concept should be explored in non-surgically managed PHF to provide on-demand analgesia prior to rehabilitation exercises to enhance compliance and improve short-term clinical outcomes.

In addition, pain was notably still the most cited reason at 3-months despite the PHF likely being mostly healed and stable at this juncture. A paper by R.F. Shah et. al. discussing upper extremity fractures has re-iterated a well-known fact about pain pathophysiology – that there are not only biological but also psychosocial determinants (e.g. poor social support, anxiety disorder) [32]. While we seek to optimise analgesia in rehabilitating PHF patients, the need for holistic patient education on PHF healing and its rehabilitation process, as well the screening and management of emotional and social health should not be overlooked. For instance, to minimize anxiety about rehabilitation-associated pain, efforts can be made to educate patients about the typical course/timeline of PHF recovery and when it should be expected/acceptable to be experiencing certain levels of pain (e.g. during rehabilitation sessions).

As such, potential arenas to expand research could be to use study designs such as randomized control trials to compare different interventions to improve PHF rehabilitation compliance. For example, nerve-block vs no nerve-block or comparing different rehabilitation motivational strategies.

### Need for further research on rehabilitation compliance

Lastly, this paper has highlighted the lack of consensus definition for rehabilitation compliance. In a 2016 systematic review, Hawley-Hague et al. identified 37 papers which discussed “exercise adherence” [22] – and showed that there is no single superior definition for rehabilitation compliance, and that what fulfils “good compliance” is often arbitrary. Majority (30/37) of papers used attendance of exercise/therapy classes as the sole measure of adherence – and high adherence was defined arbitrarily by its authors (e.g. some defined high adherence as 66.7–100% attendance, others 90–100%). Other less commonly identified measures used were duration adherence in 12/37 papers (i.e. patient-reported duration of prescribed exercises at home) and intensity adherence in 5/37 (e.g. maintenance of a certain percentage of maximum heart rate during exercise), with some papers arbitrarily defining high intensity as maintenance of 60–80% of maximal heart rate. In our paper, rather than using a single measure, a wide range of rehabilitation compliance measures were used to quantify and qualify “rehabilitation compliance” from perspectives of all stakeholders (e.g. therapist, patient). In addition to self-developed measures, the well-validated SIRAS was also adapted within the questionnaire to lend further credibility to the measurement of rehabilitation compliance. Unlike the author-developed patient-reported rehabilitation compliance measures used in the questionnaire, SIRAS is a well-validated scoring instrument for rehabilitation of musculoskeletal injuries and also has rater-agreement index values of up to 0.95 [25], minimising variability due to subjectivity and improving the score’s reproducibility. SIRAS has also been shown to have significant positive correlations with adherence to home-based exercises for rehabilitation specifically in musculoskeletal injuries [33], thereby improving the reliability of reporting of compliance for unsupervised home-based rehabilitation exercises.

Importantly, our results have shown good correlation between the author-developed rehabilitation compliance measures (patient-reported subjective compliance and average regime in prescribed exercises) and the well-validated SIRAS. Currently, there is a lack of established rehabilitation compliance measures for musculoskeletal conditions. Having targeted, easy-to-administer and validated questionnaires on musculoskeletal rehabilitation compliance could be an area of focus to (1) help develop research in the area and (2) be used in clinical practice.

### Strengths and limitations

To the authors’ knowledge, this paper is the largest study in this area – investigating the relationship between rehabilitation compliance and clinical outcomes of non-surgically treated PHFs, including short (3-month) and long-term (1-year) results for discussion. A key strength of this paper is a comprehensive set of rehabilitation compliance measures that sought to quantify/qualify rehabilitation compliance from the perspective of all stakeholders – including both patient-reported and therapist-reported measures with subjective and objective components. Lastly, linear regression modelling was used to control and account for other confounding variables which may affect the results.

Unfortunately, given the immaturity of research in rehabilitation compliance, certain measures chosen were well-validated (e.g. SIRAS) while other aspects (e.g. patient subjective compliance) were not. Furthermore, clinical outcomes up to 1-year were discussed in this paper, the authors would have liked to include rehabilitation compliance data past the 3-month mark – unfortunately, this had multiple logistical issues as most of the patients were discharged from therapy follow-up after 3 months. Whether or not these patients continued to be compliant to home-based exercises is unknown and could very well affect long-term outcomes.

Further, in terms of patient selection, while the authors tried to ensure as homogenous a study sample as possible via the inclusion/exclusion criteria, the current lack of consensus for surgical/non-surgical indications of PHFs has made doing so difficult. Without an established gold standard for this, this limitation will likely remain for all research done in this arena.

## Conclusion

This study has shown that rehabilitation compliance can predict clinical outcomes in non-surgically managed PHFs, with stronger predictive value on short-term outcomes compared to long-term outcomes. Furthermore, this study has also highlighted the need for future research in terms of rehabilitation compliance measures. Patient-reported measures used have shown some promise, with good correlation to validated and established measures such as the SIRAS. Lastly, optimising analgesia and minimizing pain can further improve rehabilitation compliance and aid outcomes – and novel techniques such as the inter-scalene block may be an area to explore to do so.

### Supplementary Information

**Additional file 1: **STROBE Statement—Checklist of items that should be included in reports of cohort studies.

**Additional file 2: Appendix 2. **Summary of Institution’s Rehabilitation Treatment Protocol for Proximal Humerus Fractures.

**Additional file 3: **Study on Proximal Humerus Evaluation of Effective tReatment (SPHEER).

## Data Availability

Data are available upon request, which can made directly to the corresponding author via email. Data includes anonymized patient demographics/injury details, clinical outcome measures, rehabilitation compliance measures.
