# Supplementary material for: Study on proximal humerus evaluation of effective treatment (SPHEER) – what is the effect of rehabilitation compliance on clinical outcomes of proximal humerus fractures
Source: BMC Musculoskelet Disord. 2023 Oct 2;24:778. doi: 10.1186/s12891-023-06894-w (PMC10544385; doi:10.1186/s12891-023-06894-w)

Appendix 2 – Summary of Institution’s Rehabilitation Treatment Protocol for Proximal Humerus Fractures


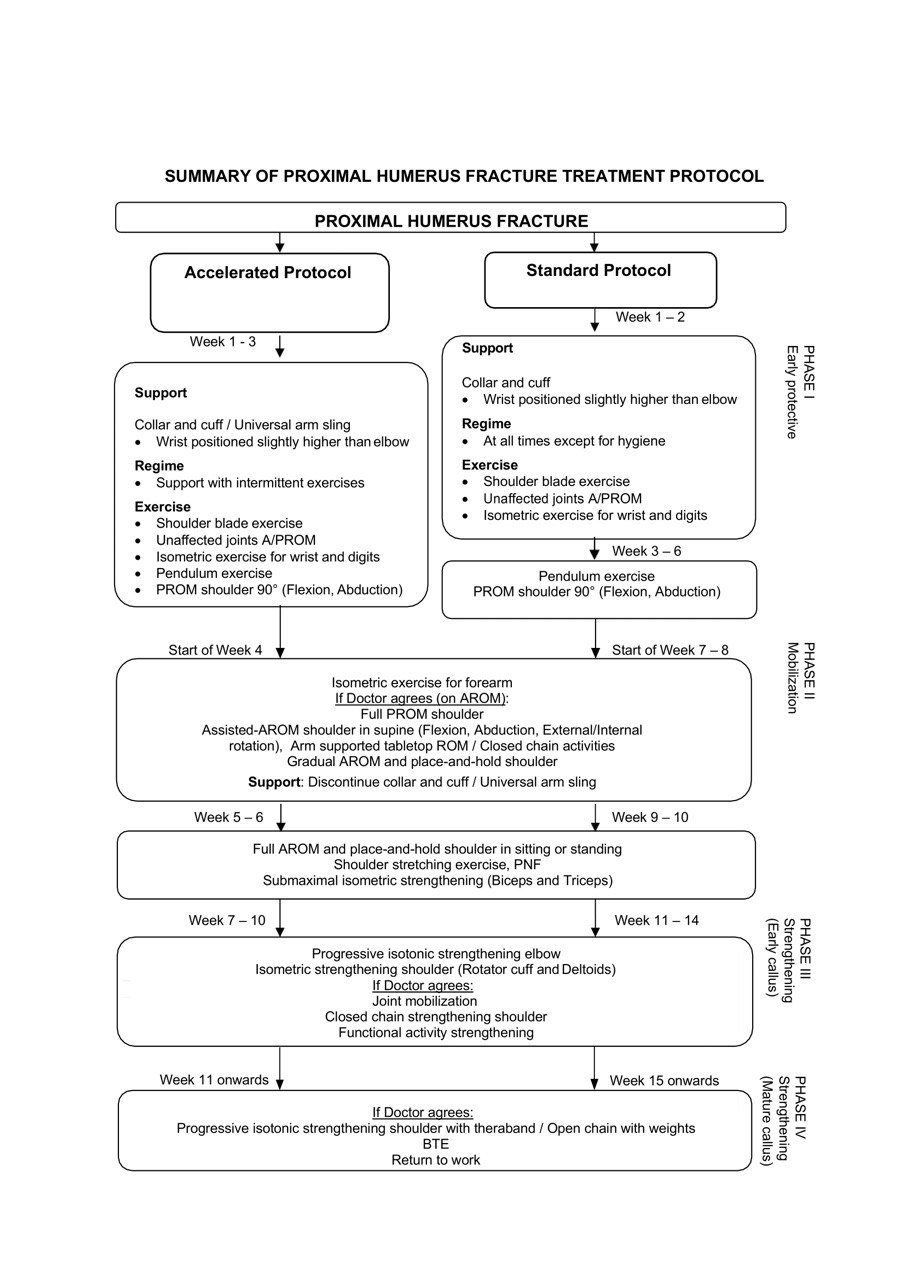

Supplement: Supplementary file 2 — Additional file 2: Appendix 2. Summary of Institution’s Rehabilitation Treatment Protocol for Proximal Humerus Fractures. [file 12891_2023_6894_MOESM2_ESM.docx]
