# Supplementary material for: Study on proximal humerus evaluation of effective treatment (SPHEER) – what is the effect of rehabilitation compliance on clinical outcomes of proximal humerus fractures
Source: BMC Musculoskelet Disord. 2023 Oct 2;24:778. doi: 10.1186/s12891-023-06894-w (PMC10544385; doi:10.1186/s12891-023-06894-w)
Supplement: Supplementary file 3 — Additional file 3: Study on Proximal Humerus Evaluation of Effective tReatment (SPHEER). [file 12891_2023_6894_MOESM3_ESM.docx]

**Study on Proximal Humerus Evaluation of Effective tReatment (SPHEER)**

Age : ­_________________________________

Gender : ☐ Male ☐ Female

Treatment type : ☐ Conservative ☐ Surgical

Protocol type : ☐ Standard ☐ Accelerated

Date of injury (DD/MM/YY) : ­_________________________________

Discharge date (DD/MM/YY) : _________________________________

Affected side : ☐ Right ☐ Left

Hand dominance : ☐ Right ☐ Left

Neer’s classification : ☐ 1 ☐ 2 ☐ 3 ☐ 4

Sling/ collar and cuff : ☐ Yes ☐ No

Objective measurement

**TO BE FILLED IN BY OCCUPATIONAL THERAPIST**

|  | Initial visit | | 6 weeks review | 3 months review | 1 year review |
| --- | --- | --- | --- | --- | --- |
|  | Date: | | Date: | Date: | Date: |
| **Average** Pain Score (0-10)  0 – no pain  10 – worse pain ever |  | |  |  |  |
| Range of motion (shoulder):   - Flex/extn (°) - Abduction (°) - IR/ER (°) | Unaffected side  /    / | Affected side  /    / | Affected side    /    / | Affected side    /    / | Affected side  /    / |
| Constant Score |  |  |  |  |  |
| Grip strength (kgF) |  |  |  |  |  |
| QuickDASH score |  | |  |  |  |

*Extn= extension ,IR= internal rotation, ER= external rotation*

*please indicate if there has been pre-existing injury to the unaffected shoulder ☐ Yes ☐ No

Final Deposition: ☐ Open date/Discharge ☐ Default

Date: _______________

No. of therapy sessions attended (till open date/discharged/default) : ______________

No. of no show/defaulted session (till open date/discharged/default): ______________

Shoulder functional AROM met : Yes*/ No

*Flex/extn : 115°/ 40° *If yes, date achieved:______________*

*Abduction : 120°*

*IR/ER : 50°/45°*

**Questionnaire on therapy program (***For patient to answer*)

**6 weeks review**

Date of administration: _________________________________

Please circle/tick/elaborate your response for the following questions. **In the last 6 weeks**

1. Rate your compliance in performing the exercises prescribed
   1. Not at all
   2. Rarely
   3. Sometimes
   4. Most of the time
   5. All the time
2. On average, what is your regime for the exercises prescribed?
   1. None at all
   2. Weekly: 1 / 2 / 3 / 4 / 5 / 6 *(please circle number of session per week)*
   3. Once a day
   4. 2-3 sessions per day
   5. > 3 sessions per day
3. Tick the reason(s) for not complying with exercises prescribed at home

*(May choose more than 1 reason)*

( ) Unsure of the exercises to do

( ) Time constraint

( ) Pain

( ) Need assistance from others

( ) Financial

( ) Forgetful

( ) Do not believe that therapy will work

( ) Others: ________________________________________

1. Currently, how often do you come for therapy review?
   1. Once a week
   2. Twice a week
   3. Once a fortnight / three weeks
   4. Once month
   5. Others:_______________________________________________

**Questionnaire on therapy program** (*For patient to answer*)

**3 months review**

Date of administration: _________________________________

Please circle/tick/elaborate your response for the following questions. **In the last 6 weeks**

1. Rate your compliance in performing the exercises prescribed
   1. Not at all
   2. Rarely
   3. Sometimes
   4. Most of the time
   5. All the time
2. On average, what is your regime for the exercises prescribed?
   1. None at all
   2. Weekly: 1 / 2 / 3 / 4 / 5 / 6 *(please circle number of session per week)*
   3. Once a day
   4. 2-3 sessions per day
   5. > 3 sessions per day
3. Tick the reason(s) for not complying with exercises prescribed at home

*(May choose more than 1 reason)*

( ) Unsure of the exercises to do

( ) Time constraint

( ) Pain

( ) Need assistance from others

( ) Financial

( ) Forgetful

( ) Do not believe that therapy will work

( ) Others: ________________________________________

1. Currently, how often do you come for therapy review?
   1. Once a week
   2. Twice a week
   3. Once a fortnight / three weeks
   4. Once month
   5. Others:_______________________________________________

**Questionnaire on therapy program** (*For therapist input*)

**6 weeks review**

Date of administration: _________________________________

1. In your opinion, rate patient compliance in exercises prescribed
   1. Not at all
   2. Slightly
   3. Moderately
   4. Very Good
   5. Extremely Good
2. In the last 6 weeks,

Circle the number that best indicates the intensity with which the patient completed the rehabilitation exercises during today appointment?


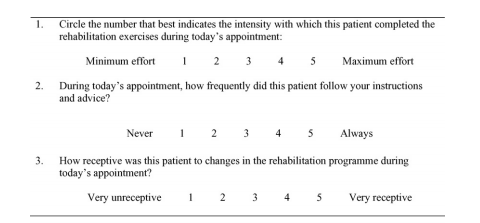


How frequently did this patient follow your instructions and advice?


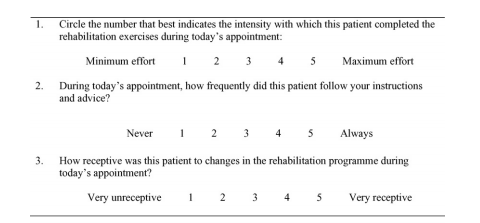


How receptive was this patient to changes in the rehabilitation program?


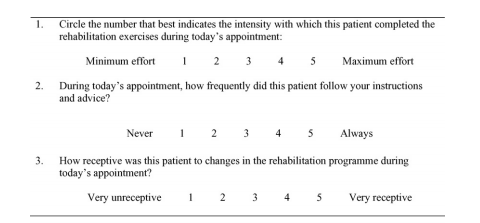


*Based on the **Sports Injury Rehabilitation Adherence Scale (SIRAS)**

1. In your opinion, what are the factors affecting patient’s compliance in home program

( ) Pain guarding

( ) Fear of re-injury

( ) Lack of interest/motivation

( ) Time constraint/Return to work

( ) Learning ability

( ) Cognitive

( ) Language

( ) Physical ability

( ) Defaulted therapy

( ) Others: ___________________________________

**Questionnaire on therapy program** (*For therapist input*)

**3 months review**

Date of administration: _________________________________

1. In your opinion, rate patient compliance in exercises prescribed
   1. Not at all
   2. Slightly
   3. Moderately
   4. Very Good
   5. Extremely Good
2. In the last 6 weeks,

Circle the number that best indicates the intensity with which the patient completed the rehabilitation exercises during today appointment?


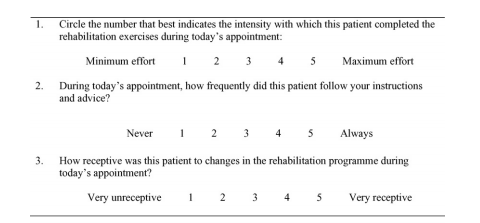


How frequently did this patient follow your instructions and advice?


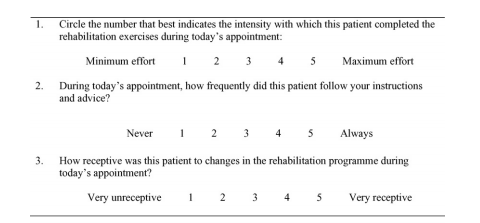


How receptive was this patient to changes in the rehabilitation program?


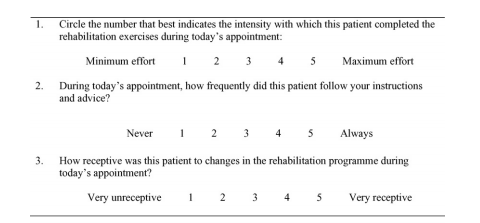


*based on the **Sports Injury Rehabilitation Adherence Scale(SIRAS)**

1. In your opinion, what are the factors affecting patient’s compliance in home program

( ) Pain guarding

( ) Fear of re-injury

( ) Lack of interest/motivation

( ) Time constraint/Return to work

( ) Learning ability

( ) Cognitive

( ) Language

( ) Physical ability

( ) Defaulted therapy

( ) Others: ___________________________________
